# Effect of lipopolysaccharide derived from surabaya isolates *of Actinobacillus actinomycetemcomitans* on alveolar bone destruction

**DOI:** 10.14202/vetworld.2018.161-166

**Published:** 2018-02-10

**Authors:** Rini Devijanti Ridwan, Sidarningsih, Tuti Kusumaningsih, Sherman Salim

**Affiliations:** 1 Department of Oral Biology, Faculty of Dental Medicine, Universitas Airlangga, Surabaya, Indonesia; 2 Department of Prosthodontic, Faculty of Dental Medicine, Universitas Airlangga, Surabaya, Indonesia

**Keywords:** *Actinobacillus actinomycetemcomitans* local isolate, lipopolysaccharide, interleukineIL-6, matrix metallopeptidase-1, receptor activator of nuclear factor kappa-Β, receptor activator of nuclear factor kappa-Β, ligand

## Abstract

**Background:**

*Actinobacillus actinomycetemcomitans’* lipopolysaccharide (LPS) has a high virulence factor. It interacts with serum protein through receptors on the epithelial cell surface, thereby increasing both interleukin (IL)-1β, and IL-6 which results in damage to periodontal tissue.

**Aim:**

The aim of the study was to identify and evaluate the effect of LPS derived from local isolates (*A. actinomycetemcomitans)* on the destruction of alveolar bone by means of several biomarkers, including; the number of osteoblasts and osteoclasts, the expression of IL-6, matrix metallopeptidase 1 (MMP-1), and receptor activator of nuclear factor kappa-Β ligand (RANKL).

**Materials and Methods:**

The isolation of LPS from *A. actinomycetemcomitans* was calculated using phenol, while purification was performed using Sephadex C-18 column chromatography. 40 Wistar rats were divided into four groups of 10. Each treatment was divided into two groups which were 0.9% NaCl and LPS induced for 7 and 14 days, respectively. Gingival and alveolar bones were further introduced into the induction area, followed by the measuring of osteoblast and osteoclast with hematoxylin-eosin staining, IL-6, MMP-1 and RANKL expression with immunohistochemical.

**Results:**

Reduced numbers of osteoblasts at the 7^th^ and 14^th^ day of treatment were detected, while those of osteoclasts increased. There was an increased expression of IL-6, MMP-1, and RANKL in the 7^th^ and 14^th^-day treatment group. Treatment of LPS from *A. actinomycetemcomitans* over 7 and 14 days resulted in damage to periodontal tissue and alveolar bone in Wistar rats.

**Conclusion:**

LPS of *A. actinomycetemcomitans* administration for 7 and 14 days causes periodontal and alveolar tissue destruction in Wistar rats.

## Introduction

Lipopolysaccharide (LPS) is a component of *A. actinomycetemcomitans* forming its cell wall as a virulence factor. LPS interacts with serum protein through receptors on the epithelial cell surface [1]. The high concentration of LPS increases interleukin (IL) - 1β and IL-6 which, in turn, results in the destruction of periodontal tissue due to a cytokine response in the epithelium, neutrophil, fibroblast, and monocyte. The process occurs through host stimulation and plays an important role in tissue damage by stimulating the macrophage to release (IL-1), IL-1β, and tumor necrosis factor (TNF). These pro-inflammatory cytokines are responsible for causing bone damage [2].

The LPS interferes with the homeostasis of collagen metabolism through collagen phagocytosis through fibroblast. A study by Shaddox *et al*. [3], found that LPS stimulates the synthesis of osteoblastic IL-1β, TNFα, IL-6, and receptor NF-кB ligand (RANKL). The virulence potential of the *A. actinomycetemcomitans* might be different. Specific Polysaccharide Antigen (SPA), serotype b of *A. actinomycetemcomitans* is responsible for the resistance mechanism to phagocytosis and kills polimorfonuclear leukocyte in humans [4]. Takahashi *et a*l. [4] reported that the SPA of serotype a and c induces IL-1 release by murine’s macrophage which is lower than the SPA of *A. actinomycetemcomitans* serotype b. Meanwhile, Patil *et al*. [5] demonstrated the higher sensitivity of various antimicrobials in other serotypes compared to that of *A. actinomycetemcomitans*. Some kinds of yeasts secrete toxins and these types called killer yeasts. These yeasts can inhibit the growth of other yeast strains and also have antimicrobial activities inhibiting growth of bacteria [6,7].

The aims of this study were to determine the effect of LPS in a local isolate of *A. actinomycetemcomitans*, as characterized by several markers of damaged alveolar, through the measuring of osteoblast and osteoclast, as well as IL-6, Matrix Metallopeptidase 1 (MMP-1) and RANKL expression.

## Materials and Methods

### Ethical approval

This study received an ethical clearance approval letter relating to human subjects from the Ethics Research Committee of Faculty of Dental Medicine, Universitas Airlangga, with number 29/KKEPK/FKG/III/2015. The research constituted Analytical Observational involving the use of an Experimental Laboratory and a cross-sectional method.

### A. actinomycetemcomitans local isolate preparation

*A. actinomycetemcomitans* was obtained from patients with aggressive periodontitis and further subcultured in Luria Berthani (Merck KgaA®, Darmstadt, Germany) for 2-3 days to obtain its morphology (visualized in 2 plates). An *A. actinomycetemcomitans* culture of 200 µl was required for four animal model groups (40 rats in total); therefore, 10 ml culture was stored at a density of 10^8^ cells every day for a period of 14 days. 200 µl LPS of *A. actinomycetemcomitans* was produced containing 200 µg/ml proteins to induce sulcus of the maxilla molar of Wistar rats over 7^th^ day to 14^th^ day. LPS was isolated and purified following Westphal and Jann’s [6] phenolic-based method. The resulting purified crude LPS and isolate were subsequently gel-filtrated in Sephadex^®^ C-18 (Sigma Aldrich™, Darmstadt, Germany) at room temperature with a disaggregation buffer as solvent (0.05 M Tris-HCl pH 9, 0.001 M EDTA, and 0.3 deoxycholate) (Sigma-Aldrich™, Darmstadt, Germany).

### Animal model study preparation

The animal model used consisted of Wistar rats divided into four groups each containing 10 rodents. Group 1 constituted a control group, induced with NaCl 0.9% for 7 days Group 2 received treatment with LPS for 7 days. Group 3 was induced with NaCl 0.9% for 14 days, while Group 4 underwent treatment with LPS for 14 days[4,5].

Treatment was injected into the sulcus of the first maxillary molar (M1) following Dumistrescu’s method [8,9]. Each group was divided into two sub-groups, one treated for 7 days, the other for 14 days. Injections of 200 µg of LPS, with a protein level of 200 µg/ml, and a density of 108 were administered for at least 7 days to obtain significant aggressive periodontitis symptoms [9]. Gingival and alveolar bones were subsequently examined into the induction area, followed by the measuring of osteoblast and osteoclast with hematoxylin-eosin (HE) staining, and IL-6, MMP-1, and RANKL expression with an immunohistochemical marker kit (Merck KgaA^®^, Darmstadt, Germany).

At the next immunohistochemical examination on glass object with 50% glycerin dropped above the glass object and viewed the results with an Electron Microscope (Automated Electron Microscope, Olympus^®^, USA). Data in normal distribution were analyzed using Shapiro-Wilk test (p>0.05).

###  Statistical analysis

Data homogeneity was analyzed using ANOVA (p<0.05). Statistical analysis was effected by means of Statistical Package for the Social Sciences (SPSS) 17.0 software for windows 8.1 by SPSS Inc., Chicago, United States.

## Results

### Measurement of osteoblast and osteoclast

The measurement of osteoblast from alveolar bones on day 7 showed data were normally distributed and homogenous with a significant value of p>0.05. ANOVA analysis showed there was a considerable difference on day 7 in the osteoblast between the treatment group 16.5±3.84 and the control group 5±0.82 (p<0.05). Measurement of osteoblast from alveolar bones carried out on day 14 showed was normally distributed and homogenous p>0.05. ANOVA analysis showed there was a marked difference in osteoblast between the treatment group 22.6±2.41 on day 14 and that of the control group 5.8±1.48 (p<0.05).

The measurement of osteoclast from alveolar bones on day 7 showed data to be normally distributed and homogenous p˂0.05. ANOVA analysis showed there was a no stable contrast in osteoclast between the treatment group on day 7 and the control group (p<0.05). The measurement of osteoclast from alveolar bones on day 14 confirmed that data were normally distributed and homogenous with a significant value of p˃0.05. ANOVA analysis showed there was a considerable difference in osteoclast between the treatment group on day 14 and the control group (p<0.05).

**Figure-1:**
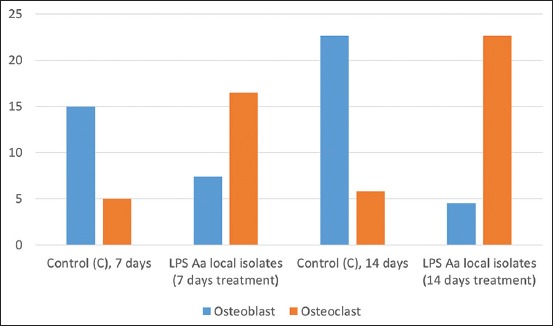
Average levels of osteoblast and osteoclast in treatment on days 7 and 14 in alveolar bone.

**Figure-2:**
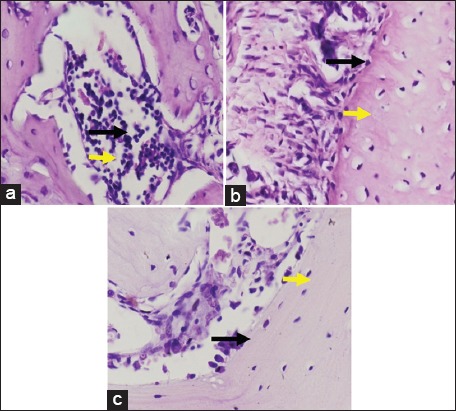
Hematoxilin-Eosin staining of gingiva tissue. Control (a); with 7-day induction of lipopolysaccharide (LPS) (b); Group with 7-day induction of LPS (c). The black arrow indicates osteoclast, while the yellow shows the location of osteoblast.

A graphic of the average alveolar osteoblast on day 7 showed 15 cells in the control group and 7.4 cells in the treatment group. The measurement of osteoclast from gingiva tissue on day 7 confirmed there to be 5 cells in the control group and 16.5 cells in the treatment group. Osteoblast measurements taken from the alveolar on day 14 showed that osteoblast represented 22.6 cells in the control group, but only 4.5 cells in the treatment group. The measurement of osteoclast-derived from gingiva tissue on day 14 established the presence of 5.8 cells in the control group, in contrast to the 22.6 cells in the treatment group. The average presence of osteoblast and osteoclast in the alveolar bone can be seen in Figure-1, while the results of HE staining of gingiva tissue are shown in Figure-2.

### Expression of IL-6, MMP-1, and RANKL

**Figure-3:**
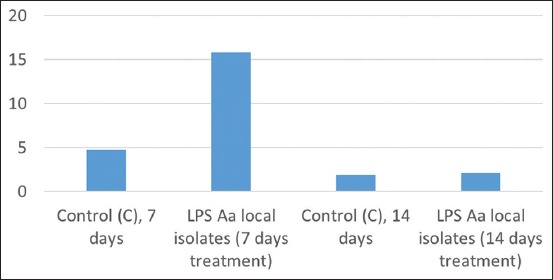
Average interleukin-6 expression in gingival tissue.

**Figure-4:**
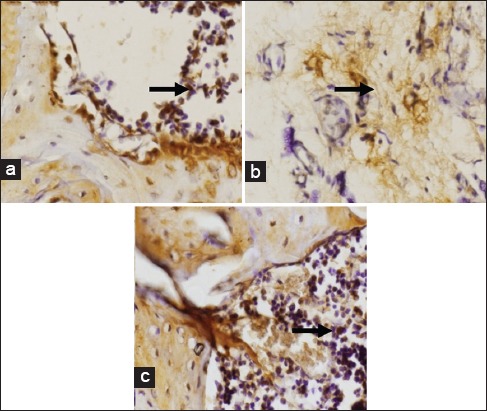
Expression of interleukin-6 in gingiva of Wistar rat. Control (a); *Actinobacillus actinomycetemcomitans* lipopolysaccharide (LPS) (day 7) (b); *A. actinomy cetemcomitans* LPS (day 14) (c), indicated by the brown regions in the nucleus (black arrow). Activated expression of interleukin -6 was shown as brown in cytoplasm of gingiva epithelium (black arrow) at 400× magnification.

The expression of IL-6 in gingival tissue during day 7 showed data were normally distributed and homogenous (p>0.05). ANOVA analysis revealed a significant difference of IL-6 expression between the day 7 treatment group 15.8±0.79 and the control group 4.7±1.16 (p<0.05). The expression of IL-6 from gingival tissue undergoing day 14 treatment showed the data to be normally distributed and homogenous (p>0.05). ANOVA analysis negated any significant difference of IL-6 expression between the day 14 treatment group 2.1±0.74 and the control group 1.9±0.74 (p<0.05). The average IL-6 expression in gingival tissue is shown in Figure-3, while the result of IHC staining of Wistar rat gingiva features in Figure-4.

**Figure-5:**
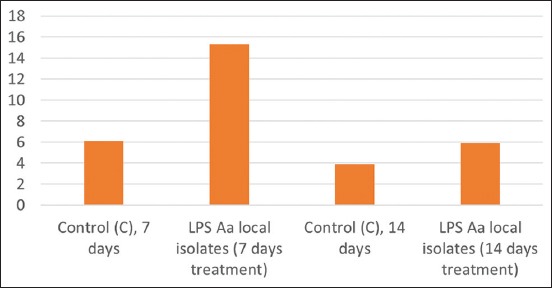
Average matrix metallopeptidase-1 expression in gingiva.

**Figure-6:**
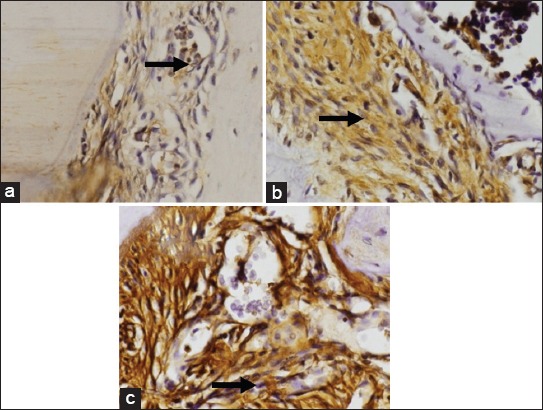
Matrix metallopeptidase-1 (MMP-1) expression in gingiva of Wistar rat. Control (a); treatment of lipopolysaccharide (LPS) (7-day) (b); treatment of LPS (14-day) (c), shown as brown in the nucleus (black arrow). Activated MMP-1 expression is highlighted as brown in cytoplasm of gingiva epithelium (black arrow) at 400× magnification.

The data of MMP-1 expression in gingiva in day 7 treatment were normally distributed and homogenous (p>0.05). ANOVA analysis confirmed a considerable difference in IL-6 expression between the day 7 treatment 15.3±0.67 and control groups 6.1±0.99 (p<0.05). The data of MMP-1 expression in gingiva during day 14 treatment were normally distributed and homogenous (p>0.05). ANOVA analysis confirmed a significant difference in IL-6 expression between the day 14 treatment group 5.9±0.74 and the control group 3.9±0.74 (p<0.05). The average MMP-1 expression in gingival tissue is shown in Figure-5, with the result of IHC staining of Wistar rat gingiva contained in Figure-6.

**Figure-7:**
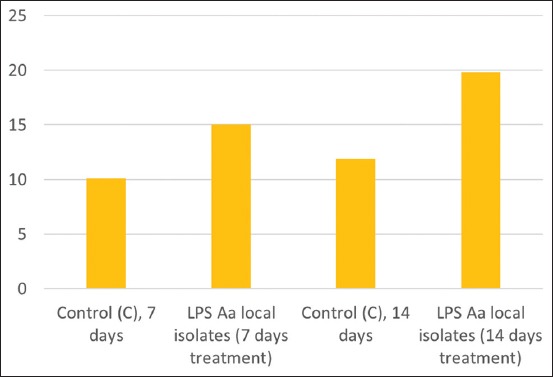
Average receptor activator of nuclear factor kappa-Β ligand expression in alveolar.

**Figure-8:**
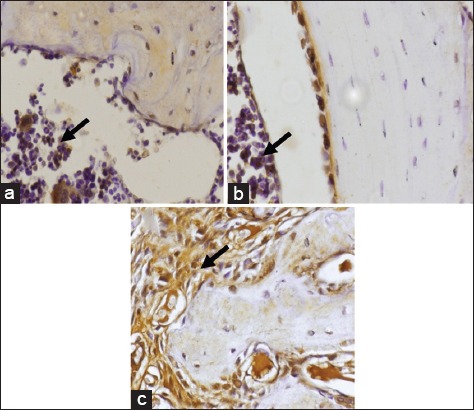
Expression of receptor activator of nuclear factor kappa-Β ligand in alveolar of Wistar rat. Control (a); *Actinobacillus actinomycetemcomitans* lipopolysaccharide (7 days) (b); *A. actinomycetemcomitans* (14 days) (c), shown as brown in the nucleus (black arrow) at a magnification of 400×.

The data of RANKL expression in gingiva after treatment lasting 7 days was normally distributed and homogenous (p>0.05). ANOVA analysis showed there was a significant difference in RANKL expression between the day 7 treatment group 15±2.5 and the control group 10.1±2.5 (p<0.05). Data relating to RANKL expression in gingiva after 14-day treatment was normally distributed and homogenous (p>0.05). ANOVA analysis showed there to be a significant difference in RANKL expression between the day 14 treatment group 18.8±1.03 and the control group 15±1.2 (p<0.05). The average RANKL expression in gingiva is shown in Figure-7 with the result of IHC staining of Wistar rat gingiva contained in Figure-8.

## Discussion

The highest concentration of osteoblasts in the alveolar bone was found in the day 14 control group and differed significantly compared to that of the treatment group. These results occurred due to the absence of aggressive periodontitis which, in contrast, was found to be present in the LPS treatment group. Osteoblast plays an important role in mineralization and hydroxyapatite precipitation by regulating calcium and phosphate levels during the composition of hydroxyapatite. Osteoblast produces alkaline phosphatase in adequate amounts in the plasma membrane responsible for bone mineralization. Osteoblast also produces cytokines such as colony stimulating factor-1, RANKL, and osteoprotegerin [8-10]. The highest concentration of alveolar osteoclast was found in the day 14 LPS treatment group and differed considerably compared to that of the control group.

The induction of LPS causes adhesion and invasion in the host. LPS is a major factor in bacteria and plays an important role in bone resorption through osteoclast stimulation in which LPS activates osteoblast to release factors attracting and activating osteoclast [11]. LPS can make macrophages activated, and can also trigger the synthesis o-f both cytokines that have pro-inflammatory activities, such as interleukin (IL)-1, IL-6, IL-8, and TNF α, as well as another cytokine, namely, IL-10 serving as regulator [2,3,12]. LPS also inhibits collagen and non-collagen protein synthesis. Referring to a study conducted by Takahashi *et al* [4] and Nair *et al*. [13] found that the LPS of *A. actinomycetemcomitans* causes bone resorption in murine calvarial by stimulating murine macrophage. *A. actinomycetemcomitans* causes alveolar bone destruction and antibody response in three rat strains; Fawn Hooded Hypersensitive, Dahl Salt Sensitive, and Norway Brown [14].

The process of periodontitis passes through 4 phases: (1) Accumulation and presence of bacteria in gingival sulcus (colonization); (2) bacterial invasion of the epithelium and gingiva; (3) host response stimulation*, acquired* and *innate* (inflammation) immune response activation; and (4) destruction of connective tissue attachment on dental enamel and bone causing irreversible damage [1]. The presence of LPS decreases osteoblast. LPS generates stimulation on RANKL which further binds to RANK, stimulating tumor necrosis receptor-associated factors-6 for osteoclast progenitor activation leading to osteoclast differentiation, activation and an increased number of osteoclasts. This, in turn, causes alveolar bone destruction which can serve as a marker if such destruction has occurred [15].

IL-6 expression in gingiva during day 7 treatment showed an increase and a marked difference compared to the control, indicating the presence of LPS in periodontal tissue. LPS stimulates pro-inflammatory cytokine activation, such as IL-6, stimulating bone resorption, and inhibiting bone formation leading to periodontal and alveolar damage [2,16].

The expression of MMP-1 in the day 7 and day 14 treatment groups showed a significant contrast compared to that of the control group. The induction of LPS *A. actinomycetemcomitans* causes pro-inflammatory cytokine stimulation. IL-8 produced by monocyte, keratinocyte, endothelial, and fibroblast cell stimulates the release of MMPs by neutrophil. One such MMP was MMP-1 which is a potential collagenase-1 and plays a role in the degradation of connective tissue in the inflamed area. The high expression of MMP-1 provokes periodontal and alveolar damage in accordance with the study conducted by Claesson *et al*. [17] that confirmed *A. actinomycetemcomitans* to be a strong stimulant to MMP-8 release, mainly due to Leukothera being one of its 15 virulence factors.

The expression of RANKL in alveolar bones tends to increase and shows a significant difference in the day 7 and day 14 treatment groups compared to the control group. The result confirms LPS-l as an antigenic component raising host immune response and further stimulating pro-inflammatory cytokines, especially TNF-α and IL-1. TNF-α and IL-1 stimulate RANKL which increase osteoclast. The high expression of osteoclast augments osteocalcin that can lead to alveolar bone resorption. A previous study conducted by Bullon *et al*. [18], posited that the increased number of osteocalcins was a marker of bone formation inhibition. An elevation osteocalcin level in serum was associated with the rate of bone destruction. Studies involving animals confirmed the role of osteocalcin in alveolar bone resorption.

## Conclusion

LPS induces IL-6 expression, osteoclastogenesis, and bone resorption. Injection of *porphyromonas gingivalis* from IL-6-removed rats causes a reduced level of bone loss. This implies that IL-6 contributes to bacteria progressivity which promotes alveolar damage. RANKL regulates the pathological and physiological conditions of bone resorption. During the pathological inflammation phase of the bone disease, RANKL expression was found to be present in B cell, T cell, and monocyte. Activation of T-cell and B-cells are cellular sources of RANKL in bone resorption in gingiva during inflammation. These results support the conclusion that LPS of *A. actinomycetemcomitans* administration over day 7 and day 14 causes periodontal and alveolar damage in Wistar rats.

## Authors’ Contributions

Conception and design of the study: RDR, TK Acquisition of data:S RDR, Sidarningsih, Analysis and/or interpretation of the data: RDR, SS. Drafting and revising the manuscript critically for important intellectual content: RDR, TK and SS. All authors read and approved the final manuscript.
